# Circumpapillary retinal nerve fiber layer thickness, anterior lamina cribrosa depth, and lamina cribrosa thickness in neovascular glaucoma secondary to proliferative diabetic retinopathy: a cross-sectional study

**DOI:** 10.1186/s12886-017-0456-9

**Published:** 2017-04-26

**Authors:** Satoshi Yokota, Yuji Takihara, Yoshihiro Takamura, Masaru Inatani

**Affiliations:** 1 Department of Ophthalmology, Faculty of Medical Sciences, University of Fukui, 23-3 Matsuokashimoaizuki, Eiheiji, Yoshida, Fukui, 910-1193 Japan; 2 Department of Ophthalmology and Visual Sciences, Kyoto University Graduate School of Medicine, Yoshidakonoe-cho, Sakyo, Kyoto, 606-8501 Japan

**Keywords:** Lamina cribrosa, Neovascular glaucoma, Optical coherence tomography, Proliferative diabetic retinopathy, Retinal nerve fiber layer thickness

## Abstract

**Background:**

To compare the lamina cribrosa between eyes with and without neovascular glaucoma (NVG) using enhanced depth imaging spectral-domain optical coherence tomography.

**Methods:**

Forty-six patients with proliferative diabetic retinopathy were enrolled in this cross-sectional study. The patients were divided into two groups based on the absence or presence of NVG (the non-NVG group and the NVG group, respectively). The intraocular pressure (IOP), circumpapillary retinal nerve fiber layer (cpRNFL) thickness, anterior lamina cribrosa depth (ALD), and laminar thickness (LT) were compared between the groups.

**Results:**

In the non-NVG group, the mean age was 66.2 ± 2.4 (mean ± standard error) years, mean maximum IOP was 18.8 ± 1.8 mmHg, mean cpRNFL thickness was 91.2 ± 3.9 μm, mean ALD was 407.0 ± 22.9 μm, and mean LT was 155.0 ± 4.7 μm. In the NVG group, the mean age was 61.4 ± 2.1 years, mean maximum IOP was 33.1 ± 1.6 mmHg, mean cpRNFL thickness was 73.6 ± 3.4 μm, mean ALD was 403.9 ± 20.1 μm, and mean LT was 156.9 ± 4.2 μm. The IOP was significantly higher and the cpRNFL was significantly thinner in the NVG group (*P* < 0.001 *and P* = 0.002, respectively). However, the age, ALD, and LT were not statistically different between the groups (*P =* 0.151, 0.919, and 0.757, respectively).

**Conclusions:**

Although the cpRNFL was thinner, the structure of the lamina cribrosa was unchanged in the NVG eyes. Axonal loss of the retinal ganglion cells in NVG patients was prior to lamina cribrosa deformation.

**Electronic supplementary material:**

The online version of this article (doi:10.1186/s12886-017-0456-9) contains supplementary material, which is available to authorized users.

## Background

The lamina cribrosa is a mesh-like structure at the optic nerve head that surrounds and protects retinal ganglion cell (RGC) axons [1–3]. The deformation and displacement of the lamina cribrosa causes a blockade of the axoplasmic flow within RGC axons [4–6]. Therefore, the lamina cribrosa is considered the primary site for axonal injury in glaucomatous optic neuropathy.

Lamina cribrosa deformation to the posterior lamina has been demonstrated in histologic studies using monkey eyes with experimental glaucoma [7]. Deepening of the anterior lamina cribrosa results from a posterior shift and/or thinning of the lamina cribrosa and causes mechanical stress on the optic nerve head.

Recent advances in optical coherence tomography (OCT) have enabled us to visualize the lamina cribrosa in clinical settings [8]. These OCT images have revealed that eyes with primary open-angle glaucoma (POAG) or exfoliation glaucoma are associated with lamina cribrosa deformations such as a deeper anterior lamina cribrosa depth (ALD) and thinner laminar thickness (LT) [9–11]. If an abnormally high intraocular pressure (IOP) was the major factor in lamina cribrosa deformation, the lamina cribrosa in secondary glaucomatous eyes should also have deeper ALDs and thinner LTs. In contrast, normal-tension glaucomatous eyes have deeper ALDs and thinner LTs than normal eyes or high-tension POAG eyes [9, 12]. The data imply that the individual vulnerability of the lamina cribrosa rather than the high IOP contributes to the deformation of the lamina cribrosa.

Neovascular glaucoma (NVG) is a severe secondary glaucoma associated with a significant risk of blindness due to uncontrolled high IOPs. Visually disturbed patients with NVG often have optic neuropathy. Actually in diabetic retinopathy patients, as the disease gets progressed, retinal nerve fiber layer thickness decreases [13]. However, to date, there has been no study to assess the association the existence of NVG and morphologic change in lamina cribrosa with OCT. If an uncontrolled high IOPs were the major factor for lamina cribrosa deformation, NVG eyes would be associated with a deeper ALD and a thinner LT similar to POAG eyes. Otherwise, only retinal nerve fiber layer thickness would decrease and ALD or LT would be unchanged.

In the present study, we compared the deformation of the lamina cribrosa in NVG eyes to non-NVG eyes in patients with proliferative diabetic retinopathy (PDR).

## Methods

### Patient selection

All study patients were examined between October 2014 and March 2016 at either the glaucoma or the diabetes service of the ophthalmology department at the University of Fukui, Fukui, Japan. Specific data from the ophthalmic examinations including slit-lamp examinations, Goldmann applanation tonometry values, fundus examinations, autorefractory meter values, and axial lengths were retrospectively obtained from the medical charts.

Patients who had been diagnosed with PDR and treated with conventional panretinal photocoagulation were included. If only one eye had NVG, that eye was analyzed. If both eyes had NVG or if neither had NVG, one of the eyes was randomly selected. Eyes with other ocular diseases that might decrease the image quality of OCT were excluded (e.g., vitreous hemorrhage). The patients were divided into two groups according to the presence of NVG by the retinal or the glaucoma specialist (Y.T. and M.I.). NVG was diagnosed when an abnormally high IOP (more than 21 mmHg) and the existence of neovascularization of the iris or angle were recorded in the medical chart during a visit for an examination. One group had PDR without NVG (non-NVG group), and the other had PDR with NVG (NVG group).

### Lamina cribrosa assessment by enhanced depth imaging spectral-domain optical coherence tomography

**Fig. 1:**
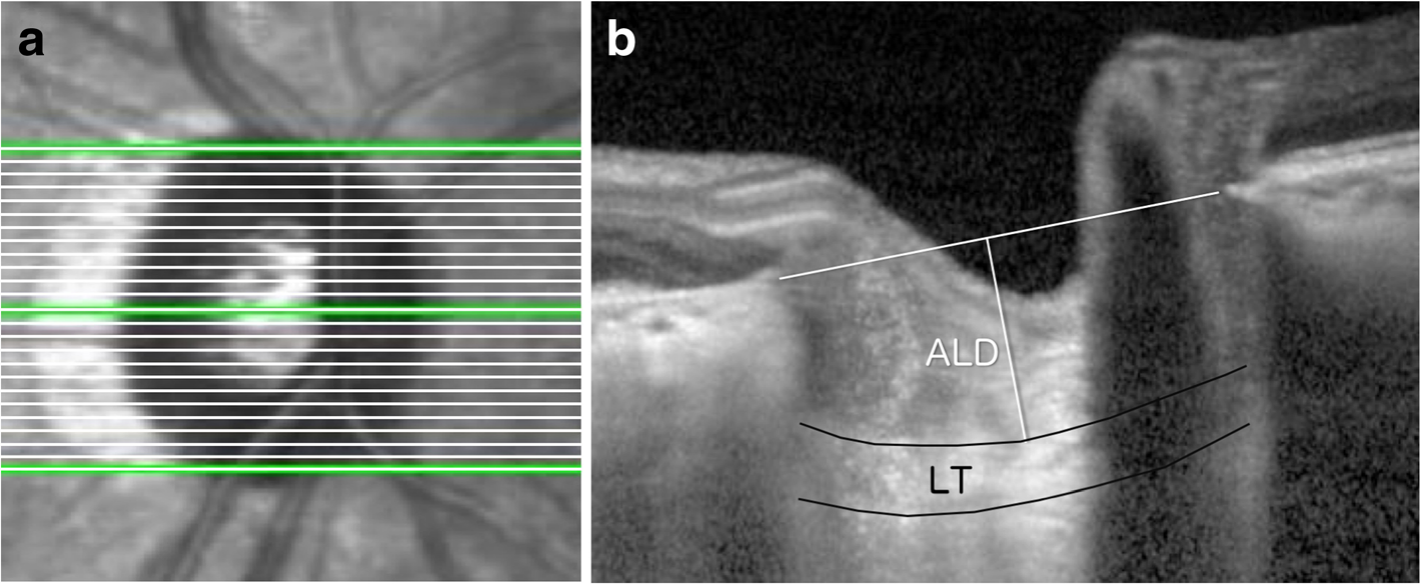
Lamina cribrosa assessment by enhanced depth imaging spectral-domain optical coherence tomography. The entire disc was subjected to horizontal B-scans with an interval of 50 μm (**a**). From these B-scans, three frames (center, mid-superior, and mid-inferior) were selected. The anterior lamina cribrosa depth was defined as the distance between the line connecting both the Bruch’s membrane opening and the anterior border of the lamina cribrosa. The laminar thickness was defined as the distance between the anterior and posterior borders of the lamina cribrosa (**b**)

OCT imaging was performed using Heidelberg Spectralis OCT (Heidelberg Engineering GmbH, Heidelberg, Germany) with an enhanced depth imaging (EDI) mode integrated into the machine. The entire optic nerve disc was subjected to horizontal B-scans at an interval of 50 μm (Fig. 1a). As described in a previous report [10], three frames (center, mid-superior, and mid-inferior) that passed through the optic nerve disc were selected from these B-scans. The ALD, which was defined as the distance between the line connecting both ends of Bruch’s membrane and the anterior border of the lamina cribrosa, was measured. The LT was defined as the distance between the anterior and posterior borders of the lamina cribrosa (Fig. 1b). The anterior and posterior borders of the lamina cribrosa were defined by a highly reflective structure below the optic cup. Both the ALD and LT were measured at the presumed vertical center of each of the three B-scans.

All measurements were obtained using the Spectralis software. All images were independently analyzed by two examiners (S.Y. and Y.T.). The mean of the two readings was adopted. To minimize the variation, the mean data of the three frames (center, mid-superior, and mid-inferior) for the ALD and the LT analyses were considered.

To evaluate the circumpapillary retinal nerve fiber layer (cpRNFL), the scan circle was positioned around the disc by experienced operators, and the image was acquired and saved. With spectral OCT, an automated computer algorithm delineated the anterior and posterior margins of the cpRNFL. The cpRNFL thickness was measured around the disc with 16 averaged circular B-scans (12 degree in a diameter); to acquire the same position, the eye movement tracking system compensated for any eye movement. The cpRNFL thickness was automatically segmented using the Spectralis software and manually corrected in case of poor segmentation.

### Statistical analyses

The baseline characteristics were compared between the NVG group and the non-NVG group. Normality was tested by Shapiro-Wilk test before using parametric tests. Parametric data were compared using Student’s t-test and non-parametric data were compared using Wilcoxon’s test. To compare categorical data, the chi-square test was used. A *P*-value of less than 0.05 was considered significant. Statistical analyses were performed using JMP Pro software version 11.0.0 (SAS Institute Inc., North Carolina).

## Results

Forty-seven patients with PDR who had previously undergone panretinal photocoagulation met the criteria for this cross-sectional study (Additional file 1: Datasets). The non-NVG and NVG groups contained 20 eyes and 26 eyes, respectively. One case in the NVG group was type 1 diabetes, and the others were type 2 or not mentioned in the medical records. The baseline data including age, gender, axial length, and spherical equivalent were not significantly different between the two groups. The maximum IOP from the medical records were statistically higher (*P <* 0.001) in the NVG group (Table 1 and Additional file 2: Table S1).

**Table 1:** Patients’ background in both groups

	Non-NVG group *n* = 20	NVG group *n* = 26	*P* value
Age (years)	66.2 ± 2.4	61.4 ± 2.1	0.151*
Gender (male/female)	14/6	20/6	0.597^†^
Axial length (mm)	23.67 ± 0.38	23.79 ± 0.33	0.819*
Spherical equivalent (D)	−1.46 ± 0.59	−2.61 ± 0.49	0.141*
Maximum IOP[range] (mmHg)	18.8 ± 1.8[14-21]	33.1 ± 1.6[23-57]	< 0.001^‡^

Numerical data was shown in mean ± standard error. * t-test, ^†^ chi-square test, ^‡^ Wilcoxon test

**Fig. 2:**
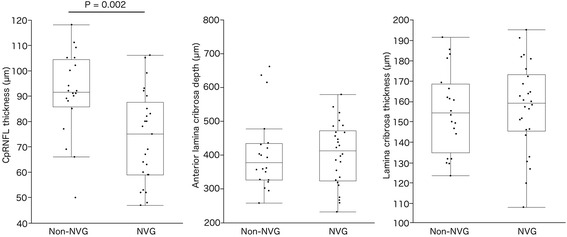
The comparison of the circumpapillary retinal nerve fiber layer (cpRNFL) thickness, anterior lamina cribrosa depth (ALD), and laminar thickness (LT) in proliferative diabetic retinopathy patients with or without neovascular glaucoma (NVG). The thickness of the cpRNFL was 91.2 ± 3.9 μm in the non-NVG group and 73.6 ± 3.4 μm in the NVG group. The ALD was 407.0 ± 22.9 μm in the non-NVG group and 403.9 ± 20.1 μm in the NVG group. The LT was 155.0 ± 4.7 μm in the non-NVG group and 156.9 ± 4.2 μm in the NVG group. The cpRNFL was significantly thinner in the NVG group (*P* = 0.002); however, there was no significant difference in the ALD (*P* = 0.919) or LT (*P* = 0.757)

The cpRNFL thickness was 91.2 ± 3.9 μm in the non-NVG group and 73.6 ± 3.4 μm in the NVG group; this demonstrated a significantly thinner cpRNFL in the NVG group (*P* = 0.002). The ALD was 407.0 ± 22.9 μm in the non-NVG group and 403.9 ± 20.1 μm in the NVG group. The LT was 155.0 ± 4.7 μm in the non-NVG group and 156.9 ± 4.2 μm in the NVG group. There were no significant differences in the ALD (*P* = 0.919) or LT (*P =* 0.757) between the two groups (Fig. 2). Among the NVG groups, cpRNFL, ALD, or LT was not different by the duration of uncontrolled high IOP (Additional file 3: Figure S1).

## Discussion

Our results showed that the cpRNFL was significantly thinner in the NVG eyes of patients with PDR. However, the ALD or LT was not different between the non-NVG and the NVG groups.

Previous reports have shown that patients with POAG or exfoliation glaucoma have thinner cpRNFLs, deeper ALDs, and thinner LTs [9–11]. Therefore, these findings were consistent with the concept that the lamina cribrosa is the primary site for glaucomatous damage because a thinner lamina cribrosa has fewer protective effects for RGC axons. However, in this study, despite the cpRNFL thickness change, there was no significant difference in the ALD or LT between the non-NVG group and the NVG group. One possible reason is that NVG secondary to PDR rapidly and highly increases the IOP compared with the slow progression associated with POAG or exfoliation glaucoma and that the rapid and high increase can damage the RGCs over the short term but might not have enough time to cause lamina cribrosa deformation. Another possible reason is that the RGCs in NVG eyes are more vulnerable to an IOP insult because of retinal ischemia or photocoagulation. The cpRNFL in diabetic patients is thinner [13]. This factor may contribute to the axonal damage of RGCs prior to lamina cribrosa deformation.

This study has several limitations. This was a cross-sectional, single hospital-based study with a small number of participants. We did not investigate the duration of the diabetes mellitus. It was possible that the cpRNFL may have become thinner due to systemic diabetic neuropathy after longer exposure to diabetes. As OCT depends on light waves, the contrast of the signals from deeper tissue was lower, even after using EDI. Therefore, the deeper border of the lamina cribrosa was highly subjective. For further analysis, a prospective study with a larger number of participants and machinery for conducting deeper tissue analysis will be necessary.

## Conclusion

In conclusion, we found that compared with cpRNFL changes, changes in the lamina cribrosa were small in the patients with NVG who had PDR. In patients with NVG, RGCs might have been more vulnerable due to retinal ischemia. This may contribute to the axonal damage of RGCs that precedes lamina cribrosa deformation.

## Additional files

Additional file 1: Datasets. These data were collected and analyzed for this paper. (XLSX 11 kb)

Additional file 2: Table S1. Patients’ comorbidities in both groups. (DOCX 23 kb)

Additional file 3: Figure S1. The comparison of the circumpapillary retinal nerve fiber layer (cpRNFL) thickness, anterior lamina cribrosa depth (ALD), and laminar thickness (LT) by the duration of uncontrolled intraocular pressures in proliferative diabetic retinopathy patients with neovascular glaucoma (NVG). The patients in the NVG group were subdivided into the two sub-groups by the duration of uncontrolled high IOPs, less than a year (shorter group) and more than a year (longer group). The thickness of the cpRNFL was 79.4 ± 4.6 μm in the shorter group and 65.6 ± 6.7 μm in the longer group. The ALD was 413.2 ± 22.0 μm in the shorter group and 377.9 ± 33.3 μm in the longer group. The LT was 155.6 ± 5.5 μm in the shorter group and 160.2 ± 8.4 μm in the longer group. There was no significant difference in the cpRNFL thickness (*P =* 0.106), the ALD (*P* = 0.388) or the LT (*P* = 0.650). (JPEG 131 kb)

## Abbreviations

ALD: Anterior Lamina cribrosa depth
cpRNFL: Circumpapillary retinal nerve fiber layer
EDI: Enhanced depth imaging
IOP: Intraocular pressure
LT: Laminar thickness
NVG: Neovascular glaucoma
OCT: Optical coherence tomography
PDR: Proliferative diabetic retionapthy
POAG: Primary open angle glaucoma
RGC: Retinal ganglion cell
