# Supplementary material for: Circumpapillary retinal nerve fiber layer thickness, anterior lamina cribrosa depth, and lamina cribrosa thickness in neovascular glaucoma secondary to proliferative diabetic retinopathy: a cross-sectional study
Source: BMC Ophthalmol. 2017 Apr 26;17:57. doi: 10.1186/s12886-017-0456-9 (PMC5407001; doi:10.1186/s12886-017-0456-9)
Supplement: Supplementary file 2 — Patients’ comorbidities in both groups. (DOCX 23 kb) [file 12886_2017_456_MOESM2_ESM.docx]

**Table S1** Patients’ comorbidities in both groups.

|  | Non-NVG group  n = 18* | NVG group  n = 23* | P value chi-square test |
| --- | --- | --- | --- |
| Hypertension | 14 (77.8%) | 18 (78.3%) | 0.970 |
| Lipid disease misbalances | 9 (50.0%) | 9(39.1%) | 0.486 |
| Disease of the circulation as heart disease | 6 (33.3%) | 7 (30.4%) | 0.843 |

*: Patients’ comorbidities were not documented in the medical records of 2 patients in the non-NVG group and 3 patients in the NVG group.
