# Supplementary figures and images for: Circumpapillary retinal nerve fiber layer thickness, anterior lamina cribrosa depth, and lamina cribrosa thickness in neovascular glaucoma secondary to proliferative diabetic retinopathy: a cross-sectional study
Source: BMC Ophthalmol. 2017 Apr 26;17:57. doi: 10.1186/s12886-017-0456-9 (PMC5407001; doi:10.1186/s12886-017-0456-9)

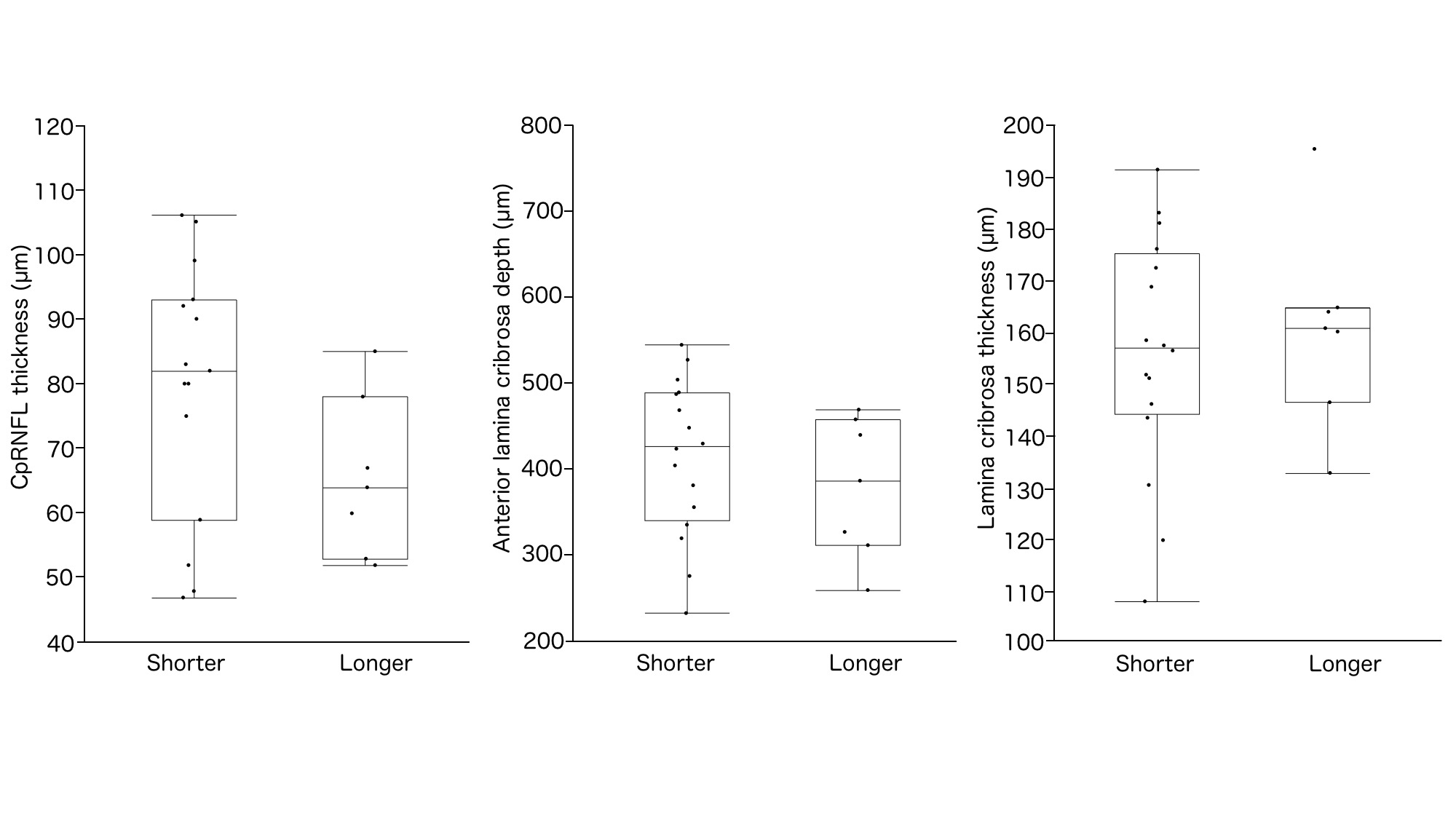

Supplement: Supplementary file 3 — The comparison of the circumpapillary retinal nerve fiber layer (cpRNFL) thickness, anterior lamina cribrosa depth (ALD), and laminar thickness (LT) by the duration of uncontrolled intraocular pressures in proliferative diabetic retinopathy patients with neovascular glaucoma (NVG). The patients in the NVG group were subdivided into the two sub-groups by the duration of uncontrolled high IOPs, less than a year (shorter group) and more than a year (longer group). The thickness of the cpRNFL was 79.4 ± 4.6 μm in the shorter group and 65.6 ± 6.7 μm in the longer group. The ALD was 413.2 ± 22.0 μm in the shorter group and 377.9 ± 33.3 μm in the longer group. The LT was 155.6 ± 5.5 μm in the shorter group and 160.2 ± 8.4 μm in the longer group. There was no significant difference in the cpRNFL thickness (P = 0.106), the ALD (P = 0.388) or the LT (P = 0.650). (JPEG 131 kb) [file 12886_2017_456_MOESM3_ESM.jpg]
